# Prognosis of pacing-dependent patients with cardiovascular implantable electronic devices

**DOI:** 10.1007/s00399-024-00996-1

**Published:** 2024-01-31

**Authors:** Wolfram Grimm, Barbara Erdmann, Kathrin Grimm, Julian Kreutz, Mariana Parahuleva

**Affiliations:** 1 Department of Cardiology, University Hospital of Marburg and Gießen, Philipps-University Marburg, Baldingerstraße, 35033 Marburg, Germany; 2 Department of Neurology, University Hospital of Erlangen, Erlangen, Germany

**Keywords:** Pacing dependency, Permanent pacemaker, Implantable cardioverter-defibrillator, Heart failure, Chronic kidney disease, Notwendigkeit der Schrittmacherstimulation, Permanenter Schrittmacher, Implantierbarer Kardioverter-Defibrillator, Herzinsuffizienz, Chronische Nierenerkrankung

## Abstract

**Background:**

Data on the prognostic significance of pacing dependency in patients with cardiovascular implantable electronic devices (CIEDs) are sparse.

**Methods:**

The prognostic significance of pacing dependency defined as absence of an intrinsic rhythm ≥ 30 bpm was determined in 786 patients with CIEDs at the authors’ institution using univariate and multivariate regression analysis to identify predictors of all-cause mortality.

**Results:**

During 49 months median follow-up, death occurred in 63 of 130 patients with pacing dependency compared to 241 of 656 patients without pacing dependency (48% versus 37%, hazard ratio [HR] 1.34; 95% confidence interval [CI]: 1.02–1.78, *P* = 0.04). Using multivariate regression analysis, predictors of all-cause mortality included age (HR 1.07; 95% CI: 1.05–1.08, *P* < 0.01), history of atrial fibrillation (HR 1.32, 95% CI: 1.03–1.69, *P* < 0.01), chronic kidney disease (HR 1.28; 95% CI: 1.00–1.63, *P* = 0.048) and New York Heart Association (NYHA) class ≥ III (HR 2.00; 95% CI: 1.52–2.62, *P* < 0.01), but not pacing dependency (HR 1.15; 95% CI: 0.86–1.54, *P* = 0.35).

**Conclusions:**

In contrast to age, atrial fibrillation, chronic kidney disease and heart failure severity as indexed by NYHA functional class III or IV, pacing dependency does not appear to be an independent predictor of all-cause mortality in patients with CIEDs.

## Abstract (de)

**Hintergrund:**

Daten zur prognostischen Relevanz der Schrittmacherabhängigkeit bei Patienten mit implantierbaren kardiovaskulären elektrischen Geräten („cardiovascular implantable electronic devices“ [CIED]) sind rar.

**Methoden:**

Die prognostische Relevanz der Schrittmacherabhängigkeit, definiert als Fehlen eines intrinsischen Rhythmus von ≥ 30 Schlägen/min, wurde bei 786 Patienten mit CIED an der Einrichtung der Autoren ermittelt. Hierfür wurden univariate und multivariate Regressionsanalysen durchgeführt, um Prädiktoren der Gesamtmortalität zu identifizieren.

**Ergebnisse:**

In einem medianen Follow-up von 49 Monaten verstarben 63 von 130 Patienten mit Schrittmacherabhängigkeit verglichen mit 241 von 656 Patienten ohne eine solche (48 % vs. 37 %, Hazard Ratio [HR] 1,34; 95 %-Konfidenzintervall [KI] 1,02–1,78, *P* = 0,04). In einer multivariaten Regressionsanalyse wurden als Prädiktoren der Gesamtmortalität identifiziert: Alter (HR 1,07; 95 %-KI 1,05–1,08, *P* < 0,01), Vorhofflimmern in der Vorgeschichte (HR 1,32, 95 %-KI 1,03–1,69, *P* < 0,01), chronische Nierenerkrankung (HR 1,28; 95 %-KI 1,00–1,63, *P* = 0,048) und New-York-Heart-Association(NYHA)-Klasse ≥ III (HR 2,00; 95 %-KI 1,52–2,62, *P* < 0,01), nicht aber die Schrittmacherabhängigkeit (HR 1,15; 95 %-KI 0,86–1,54, *P* = 0,35).

**Schlussfolgerungen:**

Anders als Alter, Vorhofflimmern, chronische Nierenerkrankung und der Schweregrad der Herzinsuffizienz (ausgedrückt durch die NYHA-Funktionsklasse III oder IV) scheint die Schrittmacherabhängigkeit kein unabhängiger Prädiktor der Gesamtmortalität von Patienten mit CIED zu sein.

## Introduction

Although several previous [1–14] studies investigated the prevalence of pacing dependency following implantation of cardiovascular implantable electronic devices (CIEDs), only two previous studies [9, 11] determined the prognostic significance of pacing dependency during follow-up. The results of these two previous studies [9, 11], however, remained inconclusive. Therefore, the purpose of the present study was to determine the prognostic significance of pacing dependency in a large cohort of 786 patients with CIEDs at the authors’ institution.

## Methods

### Study population

**Fig. 1:**
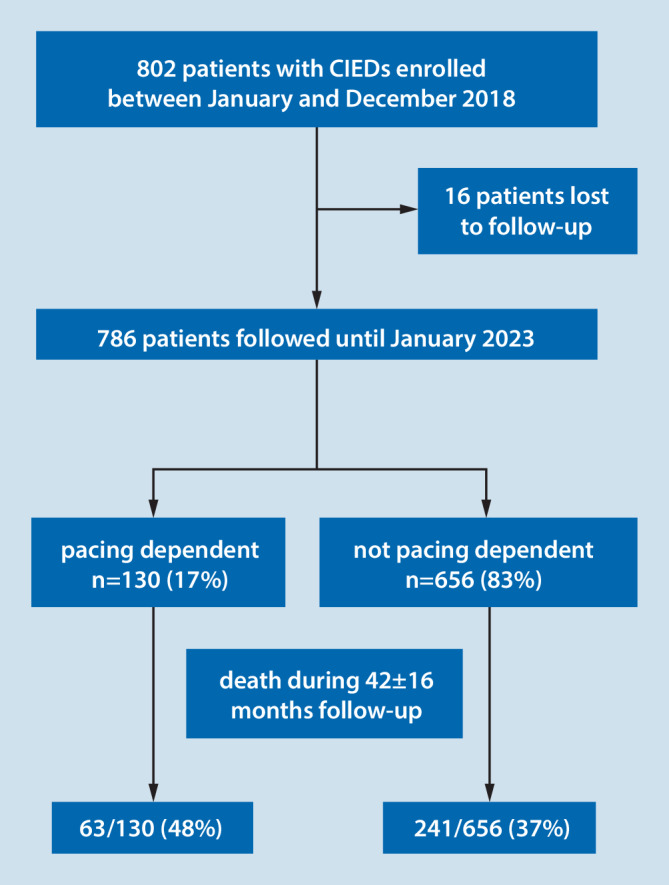
Study flow chart

The study population consisted of 786 patients with a permanent pacemaker or with an implantable defibrillator who were enrolled in the authors’ pacemaker and defibrillator outpatient clinic between January 2018 and December 2018 and who were followed until January 2023 (Fig. 1). Definitions used in this study and baseline characteristics of the study population stratified for patients with and without pacing dependency have been previously published [2]. Briefly, pacemaker dependency was defined as absence of an intrinsic rhythm ≥ 30 bpm after lowering the pacing rate to 30 bpm for at least 10 s or after transient inhibition of pacemaker therapy [2]. Chronic kidney disease of at least stage 3 was diagnosed in the presence of at least two estimated glomerular filtration rates (eGFR) using the Modification of Diet in Renal Disease formula below 60 ml/min per 1.73 m^2^ with an interval of at least 3 months. The study protocol was reviewed and approved by the ethics committee of the Philipps-University of Marburg, Germany.

### Statistical analysis

Results are expressed as mean ± standard deviation for continuous variables with normal distribution and median values with interquartile range (IQR) for continuous variables without normal distribution. Univariate comparisons of clinical characteristics between patients with and without pacemaker dependency were performed using Student’s t‑test or Mann-Whitney U test for continuous variables, and categorical values were compared using chi-square and Fisher’s exact tests where appropriate. Univariate and multivariate Cox regression analysis was used to generate a multivariate model including all potential predictors of all-cause mortality during follow-up listed in Tables 1 and 2. All probability values reported are two-sided, and a probability value of *P* < 0.05 was considered to indicate statistical significance. SPSS software version 29 (IBM, Armonk, NY, USA) was used for all statistical analyses.

**Table 1:** Clinical characteristics of 786 patients with and without pacing dependency

	All patients	Pacing dependency		*P* value
Clinical variable	*n* = 786	Yes (*n* = 130)	No (*n* = 656)	
Age, years	74 ± 13	75 ± 12	74 ± 13	0.30
Male gender, *n* (%)	512 (65)	88 (68)	424 (65)	0.57
Arterial hypertension, *n* (%)	612 (78)	104 (80)	508 (77)	0.52
Diabetes mellitus, *n* (%)	142 (18)	19 (15)	123 (19)	0.32
Atrial fibrillation before implant, *n* (%)	238 (30)	37 (28)	201 (31)	0.70
Chronic kidney disease, *n* (%)	326 (41)	69 (53)	257 (39)	< 0.01
*Heart failure severity*				
Left ventricular ejection fraction, %	43 ± 13	40 ± 12	44 ± 13	< 0.01
Left ventricular ejection fraction ≤ 30%, *n*	175 (22)	40 (31)	135 (21)	0.01
NYHA functional class III or IV, *n*	314 (40)	73 (56)	241 (37)	< 0.01
*Underlying cardiac disease, n (%)*				
Coronary artery disease	310 (39)	45 (35)	265 (40)	0.22
Nonischemic dilated cardiomyopathy	115 (15)	25 (19)	90 (14)	0.14
Hypertensive heart disease	176 (22)	35 (27)	141 (21)	0.18
Valvular heart disease	100 (13)	17 (13)	83 (13)	0.89
Other cardiac diseases ^a^	12 (2)	4 (3)	8 (1)	0.24
No structural heart disease	73 (9)	4 (3)	69 (11)	0.01
*Previous cardiac surgery, n (%)*				
Aortocoronary bypass grafting	95 (12)	17 (13)	78 (12)	0.82
Surgical aortic valve replacement	29 (4)	4 (3)	25 (4)	0.88
Transcatheter aortic-valve replacement, *n* (%)	43 (5)	9 (7)	34 (5)	0.56
*Cardiovascular implantable electronic device, n (%)*				
Antibradycardia pacemaker	555 (71)	103 (79)	452 (69)	0.02
Implantable cardioverter-defibrillator	231 (29)	27 (21)	204 (31)	
Cardiac resynchronisation therapy device	92 (12)	28 (22)	64 (10)	0.01
*Indication for CIED implantation, n (%)*				
Sick sinus syndrome	191 (24)	12 (9)	179 (27)	< 0.01
Second or third-degree AV block	244 (31)	95 (73)	149 (23)	< 0.01
Atrial fibrillation with bradycardia	124 (16)	14(11)	110 (17)	0.11
Carotid sinus syndrome	1 (0.1)	0 (0)	1 (0.2)	0.66
Prophylactic ^b^	226 (29)	9 (7)	217 (33)	< 0.01
Amount of ventricular pacing, %	46 ± 30	98 ± 12	36 ± 41	< 0.01
*Medication*				
β‑Blockers	530 (67)	84 (65)	446 (68)	0.45
ACE inhibitors or ARBs	580 (74)	101 (77)	479 (73)	0.32
Diuretics	538 (68)	99 (76)	439 (67)	0.04
Aldosterone antagonists	219 (28)	27 (21)	192 (29)	0.06
Angiotensin-neprilysin inhibitor	25 (3)	1 (1)	24 (4)	0.15

Values are given as mean ± SD for continuous variables, and numbers and percentages for categorical variables, unless specified otherwise

^a^ Other cardiac diseases include hypertrophic cardiomyopathy, cardiac sarcoidosis, cardiac amyloidosis, and tricuspid valve replacement

^b^ Implantable defibrillator without symptomatic bradyarrhythmia at implant

*ACE* angiotensin converting enzyme, *ARB* angiotensin receptor blocker, *AV* atrioventricular, *CIED* cardiovascular implantable electronic device, *NYHA* New York Heart Association

**Table 2:** Univariate predictors of mortality in 786 patients with and without pacing dependency

	All patients	Death during follow-up		Univariate regression analysis	
Clinical variable	*n* = 786	Yes (*n* = 304)	No (*n* = 482)	*P* value	HR (95% CI)
Pacing dependency, *n* (%)	130 (17)	63 (21)	67 (14)	0.04	1.35 (1.02–1.78)
Amount of ventricular pacing, %	46 ± 30	55 ± 43	41 ± 44	< 0.01	1.01 (1.00–1.01)
Age, years	74 ± 13	80 ± 8	70 ± 14	< 0.01	1.08 (1.06–1.09)
Male gender, *n* (%)	512 (65)	126 (41)	326 (68)	0.02	0.76 (0.61–0.93)
Arterial hypertension, *n* (%)	612 (78)	270 (89)	342 (71)	< 0.01	2.52 (1.78–3.60)
Diabetes mellitus, *n* (%)	142 (18)	63 (21)	79 (16)	0.21	1.20 (0.91–1.60)
Atrial fibrillation before implant, *n* (%)	238 (30)	127 (42)	111 (23)	< 0.01	2.02 (1.61–2.54)
Chronic kidney disease, *n* (%)	326 (41)	173 (57)	153 (32)	< 0.01	2.67 (1.81–2.85)
*Heart failure severity*					
Left ventricular ejection fraction ≤ 30%	175 (22)	86 (28)	89 (18)	< 0.01	1.44 (1.12–1.85)
NYHA functional class III or IV	314 (40)	181 (60)	133 (28)	< 0.01	2.75 (2.12–3.46)
*Underlying cardiac disease, n (%)*					
Coronary artery disease	310 (39)	139 (46)	171 (35)	0.02	1.30 (1.04–1.63)
Nonischemic dilated cardiomyopathy	115 (15)	27 (9)	88 (18)	< 0.01	0.51 (0.34–0.76)
Hypertensive heart disease	176 (22)	77 (25)	99 (21)	0.13	1.22 (0.94–1.56)
Valvular heart disease	100 (13)	44 (14)	56 (12)	0.20	1.23 (0.89–1.70)
Other cardiac diseases	12 (2)	2 (1)	10 (2)	0.23	0.43 (0.11–1.72)
No structural heart disease	73 (9)	15 (5)	58 (12)	< 0.01	0.50 (0.30–0.85)
*Previous cardiac surgery, n (%)*					
Aortocoronary bypass grafting	95 (12)	41 (13)	54 (11)	0.48	1.13 (0.81–1.56)
Surgical aortic valve replacement	29 (4)	8 (3)	21 (4)	0.24	0.65 (0.32–1.32)
Transcatheter aortic-valve replacement	43 (5)	20 (7)	23 (5)	0.19	1.36 (0.86–2.14)
*Cardiovascular implantable electronic device, n (%)*					
Antibradycardia pacemaker	555 (71)	230 (76)	325 (67)	0.02	1.37 (1.05–1.78)
Implantable cardioverter-defibrillator	231 (29)	74 (24)	157 (33)	0.02	0.73 (0.56–0.95)
Cardiac resynchronisation therapy device	92 (12)	39 (13)	53 (11)	0.63	1.09 (0.78–1.52)
*Indication for CIED implantation, n (%)*					
Sick sinus syndrome	191 (24)	74 (24)	117 (24)	0.71	1.05 (0.81–1.37)
Second or third-degree AV block	244 (31)	95 (31)	149 (31)	0.63	0.94 (0.74–1.20)
Atrial fibrillation with bradycardia	124 (16)	68 (22)	56 (12)	< 0.01	1.78 (1.36–2.33)
Carotid sinus syndrome	1 (0.1)	0 (0)	1 (0.2)	0.64	–
Prophylactic ^a^	226 (29)	67 (22)	159 (33)	< 0.01	0.66 (0.50–0.86)
*Medication, n (%)*					
β‑Blockers	530 (67)	217 (71)	313 (65)	0.03	1.32 (1.03–1.70)
ACE inhibitors or ARBs	580 (74)	213 (70)	367 (76)	0.02	0.74 (0.58–0.94)
Diuretics	538 (68)	248 (82)	290 (60)	< 0.01	2.35 (1.76–3.14)
Aldosterone antagonists	219 (28)	85 (28)	134 (28)	0.94	1.01 (0.79–1.30)
Angiotensin-neprilysin inhibitor	25 (3)	11 (4)	14 (3)	0.54	1.21 (0.66–2.21)

Values are given as mean ± SD for continuous variables, and numbers and percentages for categorical variables

^a^ Implantable defibrillator without symptomatic bradyarrhythmia at implant

*ACE* angiotensin converting enzyme, *ARB* angiotensin receptor blocker, *AV* atrioventricular, *CIED* cardiovascular implantable electronic device, *NYHA* New York Heart Association

## Results

### Clinical characteristics

The clinical characteristics of 786 study patients are summarized in Table 1 stratified for patients with and without pacing dependency at enrollment. A total of 130 patients (17%) were found to be pacing-dependent, while 656 patients (83%) were not pacing-dependent. The majority of patients were male (65%). Mean age at device implant was 74 ± 13 years. Indication for pacemaker implantation was high-degree atrioventricular (AV) block in 244 patients (31%), sick sinus syndrome in 191 patients (24%), carotid sinus syndrome in one patient (0.1%), and atrial fibrillation with bradycardia in 124 patients (16%). The remaining 232 patients (29%) had no indication for antibradycardia pacing at the time of implantable defibrillator implantation for primary or secondary prevention of sudden death.

### Predictors of all-cause mortality during follow-up

**Fig. 2:**
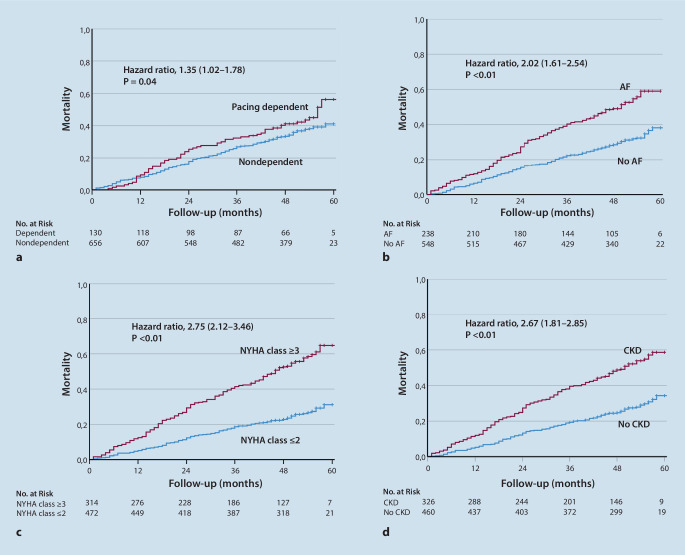
Kaplan-Meier curves for all-cause mortality stratified for: **a** pacing-dependent patients versus nondependent patients; **b** patients with a history of atrial fibrillation (*AF*) versus patients without a history of atrial fibrillation; **c** patients with New York Heart Association (*NYHA*) class III or IV versus patients with NYHA class I or II; **d** patients with chronic kidney disease (*CKD*) versus patients without chronic kidney disease

The mean duration of follow-up was 42 ± 16 months (median 49 months; interquartile range 33–53 months). Death during follow-up occurred in 63 of 130 patients with pacing dependency compared to 241 of 656 patients without pacing dependency (48% versus 37%, hazard ratio [HR] 1.34; 95% confidence interval [CI]: 1.02–1.78, *P* = 0.04). The results of univariate regression analysis of potential predictors of all-cause mortality are summarized in Table 2 and Kaplan-Meier survival curves are shown in Fig. 2. Univariate predictors of all-cause mortality included pacing dependency, age, female gender, arterial hypertension, history of atrial fibrillation, chronic kidney disease, left ventricular ejection fraction ≤ 30%, New York Heart Association (NYHA) functional class III or IV, the presence of coronary artery disease, the need for diuretics and the lack of ACE inhibitor or angiotensin blocker therapy (Table 2).

The results of multivariate regression analysis of potential predictors of all-cause mortality are summarized in Table 3. Multivariate predictors of all-cause mortality included age (HR 1.07; 95% CI: 1.05–1.08, *P* < 0.01), history of atrial fibrillation (HR 1.32, 95% CI: 1.03–1.69, *P* < 0.01), chronic kidney disease (HR 1.28; 95% CI: 1.00–1.63, *P* = 0.048), and NYHA class ≥ III (HR 2.00, 95% CI: 1.52–2.62, *P* < 0.01). Pacing dependency was not a significant predictor of all-cause mortality using multivariate analysis (HR 1.15, 95% CI: 0.86–1.54, *P* = 0.35).

**Table 3:** Multivariate predictors of mortality in 786 patients with and without pacing dependency

	All patients	Death during follow-up		Multivariate Cox analysis	
Clinical variable	*n* = 786	Yes (*n* = 304)	No (*n* = 482)	*P* value	HR (95% CI)
Pacing dependency, *n* (%)	130 (17)	63 (21)	67 (14)	0.35	1.15 (0.86–1.54)
Age, years	74 ± 13	80 ± 8	70 ± 14	< 0.01	1.07 (1.05–1.08)
History of atrial fibrillation, *n* (%)	238 (30)	127 (42)	111 (23)	0.026	1.32 (1.03–1.69)
Chronic kidney disease, *n* (%)	326 (41)	173 (57)	153 (32)	0.048	1.28 (1.00–1.63)
NYHA functional class III or IV	314 (40)	181 (60)	133 (28)	< 0.01	2.00 (1.52–2.62)

Values are given as mean ± SD for continuous variables, and numbers and percentages for categorical variables

*HR (95% CI)* hazard ratio (95% confidence interval), *NYHA* New York Heart Association

## Discussion

The main finding of the present study is that in the authors’ cohort of 786 patients with CIEDs, independent predictors of all-cause mortality include age, history of atrial fibrillation, chronic kidney disease and heart failure severity as indexed by NYHA functional class III or IV, but not pacing dependency. Their findings suggest that pacing dependency is merely a marker, but not a predictor for all-cause mortality in patients with CIEDs.

Several previous investigators [1–14], including the authors’ previous report [2], found a significant association between pacemaker dependency in patients with CIED and second or third degree AV block at implant, age, male gender and heart failure severity as indexed by a higher NYHA functional class, reduced left ventricular ejection fraction and elevated brain natriuretic peptide. In addition, a twofold risk for pacemaker dependency in patients with CIED and chronic kidney disease compared to patients without chronic kidney disease was found [2]. Due to the lack of follow-up data, however, most previous studies [2–8, 10, 12–14] investigating the prevalence of pacing dependency in patients with CIEDs did not provide information on whether pacing dependency is an independent prognostic predictor in patients with CIEDs or merely a marker for more advanced heart disease and comorbidities including heart failure and chronic kidney disease.

More than two decades ago, the Dual Chamber and VVI Implantable Defibrillator (DAVID) trial [15] showed that in selected patients with no indication for cardiac pacing and a reduced left ventricular ejection fraction of 40% or less, frequent right ventricular pacing had a detrimental prognostic effect by increasing the combined endpoint of death or hospitalization for heart failure. Furthermore, Kiehl et al. [16] described an increased rate of pacing-induced cardiomyopathy also in patients with preserved left ventricular ejection fraction at pacemaker implant in the presence of a right ventricular pacing burden of at least 20% [16]. Subsequently, Khurshid et al. [17] demonstrated that pacing induced-cardiomyopathy could be reversed in the majority of patients by upgrading the pacing system to cardiac resynchronization therapy. In the present study, pacing-dependent patients had a mean amount of ventricular pacing of 98% compared to 36% ventricular pacing in patients without pacing dependency. Despite this high amount of ventricular pacing in pacing-dependent patients, pacing dependency failed to predict all-cause mortality using multivariate analysis in the present study. Raza et al. [9] observed the need for permanent pacemaker implantation for high-degree AV block (55%) or bradycardia (45%) in 141 of 6268 patients after cardiac surgery with a prevalence of pacemaker dependency of 40% in paced patients. Similar to the present study, the mean amount of ventricular pacing was much higher in pacing-dependent patients (91%) compared to nondependent patients (51%). During 5.6-year mean follow-up, Raza et al. [9] found a significant association between permanent pacemaker requirement after surgery and subsequent mortality by univariate analysis but not by multivariate analysis. Of note, Raza et al. [9] compared only the outcomes of patients with and without the need for a permanent pacemaker after surgery. In contrast to the present study, Raza et al. [9] did not perform a subgroup analysis of pacemaker patients with versus without pacing dependency. Sood et al. [11] investigated the prevalence and prognostic significance of pacing dependency in 1058 patients who received an implantable cardioverter-defibrillator for primary or secondary prevention of sudden cardiac death during 4.2 years mean follow-up.

Similar to the findings of the authors’ study, Sood et al. [11] found pacing dependency to be associated with older age and a history of atrial fibrillation during follow-up. In contrast to the present study, Sood et al. [11] found pacing dependency to also be associated with a 48% increased risk for all-cause mortality using multivariate analysis, whereas pacing dependency was associated with a 35% increased mortality only by univariate analysis but not by multivariate analysis in the present study. This discrepancy between the study by Sood et al. [11] and this study may in part be explained by differences in study protocol and patient population. First, Sood et al. [11] defined pacemaker dependency as an intrinsic rhythm < 40 beats per minute after inhibiting the pacemaker or an intrinsic rhythm < 50 bpm with transient symptoms of dizziness, whereas pacemaker dependency in the present study was defined as absence of an intrinsic rhythm ≥ 30 bpm after lowering the pacing rate to 30 bpm for at least 10 s or after transient inhibition of pacemaker therapy. Secondly, Sood et al. [11] exclusively investigated patients with implantable defibrillators with a mean left ventricular ejection fraction of 30%, whereas the majority of patients in the present study received permanent antibradycardia pacemakers with a significantly higher mean left ventricular ejection fraction of 43%. Finally, multivariate analysis in this study also included comorbidities like arterial hypertension, diabetes mellitus, chronic kidney disease and previous cardiac surgery or transcatheter aortic valve replacement. In the authors’ previous report [2] describing the baseline characteristics of pacing-dependent versus nondependent patients, they already found a twofold risk for pacing dependency in patients with CIEDs and chronic kidney disease. Their present follow-up report demonstrates that chronic kidney disease but not pacing dependency is an independent predictor of all-cause mortality in patients with CIEDs in addition to older age, history of atrial fibrillation and NYHA functional heart failure class III or IV.

## Conclusions

In contrast to age, history of atrial fibrillation, chronic kidney disease and heart failure severity as indexed by NYHA functional class III or IV, pacing dependency does not appear to be an independent predictor of all-cause mortality in patients with CIEDs.

## Declarations

For this article no studies with human participants or animals were performed by any of the authors. All studies mentioned were in accordance with the ethical standards indicated in each case.
